# The Complete Mitochondrial Genome of *Galba pervia* (Gastropoda: Mollusca), an Intermediate Host Snail of *Fasciola* spp

**DOI:** 10.1371/journal.pone.0042172

**Published:** 2012-07-26

**Authors:** Guo-Hua Liu, Shu-Yan Wang, Wei-Yi Huang, Guang-Hui Zhao, Shu-Jun Wei, Hui-Qun Song, Min-Jun Xu, Rui-Qing Lin, Dong-Hui Zhou, Xing-Quan Zhu

**Affiliations:** 1 State Key Laboratory of Veterinary Etiological Biology, Key Laboratory of Veterinary Parasitology of Gansu Province, Lanzhou Veterinary Research Institute, Chinese Academy of Agricultural Sciences, Lanzhou, Gansu Province, China; 2 College of Veterinary Medicine, Hunan Agricultural University, Changsha, Hunan Province, China; 3 College of Animal Science and Technology, Guangxi Univesity, Nanning, Guangxi Zhuang Nationality Autonomous Region, China; 4 College of Veterinary Medicine, Northwest A & F University, Yangling, Shaanxi Province, China; 5 Institute of Plant and Environmental Protection, Beijing Academy of Agriculture and Forestry Sciences, Beijing, China; 6 Laboratory of Parasitology, College of Veterinary Medicine, South China Agricultural University, Guangzhou, Guangdong Province, China; 7 College of Animal Science and Technology, Yunnan Agricultural University, Kunming, Yunnan Province, China; Macquarie University, Australia

## Abstract

Complete mitochondrial (mt) genomes and the gene rearrangements are increasingly used as molecular markers for investigating phylogenetic relationships. Contributing to the complete mt genomes of Gastropoda, especially Pulmonata, we determined the mt genome of the freshwater snail *Galba pervia*, which is an important intermediate host for *Fasciola* spp. in China. The complete mt genome of *G. pervia* is 13,768 bp in length. Its genome is circular, and consists of 37 genes, including 13 genes for proteins, 2 genes for rRNA, 22 genes for tRNA. The mt gene order of *G. pervia* showed novel arrangement (tRNA-His, tRNA-Gly and tRNA-Tyr change positions and directions) when compared with mt genomes of Pulmonata species sequenced to date, indicating divergence among different species within the Pulmonata. A total of 3655 amino acids were deduced to encode 13 protein genes. The most frequently used amino acid is Leu (15.05%), followed by Phe (11.24%), Ser (10.76%) and IIe (8.346%). Phylogenetic analyses using the concatenated amino acid sequences of the 13 protein-coding genes, with three different computational algorithms (maximum parsimony, maximum likelihood and Bayesian analysis), all revealed that the families Lymnaeidae and Planorbidae are closely related two snail families, consistent with previous classifications based on morphological and molecular studies. The complete mt genome sequence of *G. pervia* showed a novel gene arrangement and it represents the first sequenced high quality mt genome of the family Lymnaeidae. These novel mtDNA data provide additional genetic markers for studying the epidemiology, population genetics and phylogeographics of freshwater snails, as well as for understanding interplay between the intermediate snail hosts and the intra-mollusca stages of *Fasciola* spp..

## Introduction

Many snails within the families Lymnaeidae and Planorbidae act as intermediate hosts of medically and veterinary important digenean trematodes that infect humans and domestic animals (especially sheep and cattle) [1], [2]. *Galba pervia* (Pulmonata: Lymnaeidae) is widely distributed and is the dominant host snail for transmission of *Fasciola* spp. in China [3]. Fascioliasis caused by *Fasciola* spp. is a signifancant disease of livestock animals causing substantial economic impact [4]–[7]. More importantly, millions of humans have been infected by *Fasciola* spp. in a number of countries [8].

The metazoan mitochondrial (mt) genome, ranging in length from 14 to 18 kb, is typically circular and usually contains 36–37 genes, including 12–13 protein-coding genes, 2 ribosomal RNA (rRNA) genes and 22 transfer RNA (tRNA) genes [9]. In addition, metazoan mt genome usually contains at least one lengthy noncoding region which is essential regulatory element for the initiation of transcription and replication [9]. Mitochondrial DNA (mtDNA) has long been extensively used as genetic markers to resolve evolutionary relationships among animal species due to their maternal inheritance, higher mutation rates than nuclear genes, and relatively conserved genome structures compared to ribosomal DNA [10]–[15].

In coelomate animals, mt gene arrangements are usually relatively stable within each phylum [16]. However, Mollusca, the second largest animal phylum, exhibit high diversity in their mt genome structures. For example, the mt genomes of *Mytilus edulis*, *Argopecten irradians* and *Chlamys farreri* contain supernumerary or lost tRNA genes [17], [18]. The Gastropoda are the largest class of the Mollusca and their mt gene arrangements also exhibit high levels of varibility [16]. There have been considerable controversies regarding the phylogenetic relationships of Gastropoda. Gastropoda were traditionally classified into three main subclasses based on their morphological characters: Prosobranchia, Opisthobranchia and Pulmonata. In modern taxonomies, Opisthobranchia and Pulmonata usually are clustered together in the clade Euthyneura [19], [20]. When mt gene rearrangements occur, they may usually provide very powerful phylogenetic information for resolving phylogenetic relationships among taxa [21]. Compared to other metazoan animals, only 21 complete mt genome sequences of Pulmonata species have been sequenced and deposited in GenBank (Table 1) to date, and only a low quality mt genome has been determined for the family Lymnaeidae [22].

**Table 1:** Mitochondrial genome sequences of Pulmonata sequenced prior to the present study.

Family	Species	Length (bp)	Accession numbers
Clausiliidae	*Albinaria coerulea*	14130	NC_001761
Volvatellidae	*Ascobulla fragilis*	14745	NC_012148
Planorbidae	*Biomphalaria glabrata*	13670	NC_005439
	*Biomphalaria tenagophila*	13722	NC_010220
Helicidae	*Cepaea nemoralis*	14100	NC_001816
Placobranchidae	*Elysia chlorotica*	14132	NC_010567
Ellobiidae	*Myosotella myosotis*	14246	NC_012434
	*Auriculinella bidentata*	14135	NC_016168
	*Ovatella vulcani*	14274	NC_016175
	*Pedipes pedipes*	16708	NC_016179
Onchidiidae	*Onchidella celtica*	14150	NC_012376
	*Platevindex mortoni*	13991	NC_013934
	*Peronia peronii*	13968	NC_016181
Pyramidellidae	*Pyramidella dolabrata*	13856	NC_012435
Siphonariidae	*Siphonaria pectinata*	14065	NC_012383
	*Siphonaria gigas*	14518	NC_016188
Veronicellidae	*Rhopalocaulis grandidieri*	14523	NC_016183
Amphibolidae	*Salinator rhamphidia*	14007	NC_016185
Succineidae	*Succinea putris*	14092	NC_016190
Lymnaeidae	*Radix balthica*	13993	HQ330989
Trimusculidae	*Trimusculus reticulatus*	14044	NC_016193

The objectives of the present study were to determine the complete mt sequence of the *G. pervia*, to compare the mt sequence with those of *Radix balthica* to infer further insights into the high variability of Gastropoda mitochondrial genomes, and to study phylogenetic relationships of Pulmonata using mt sequence dataset.

## Results and Discussion

### Genome content and organization

**Figure 1:**
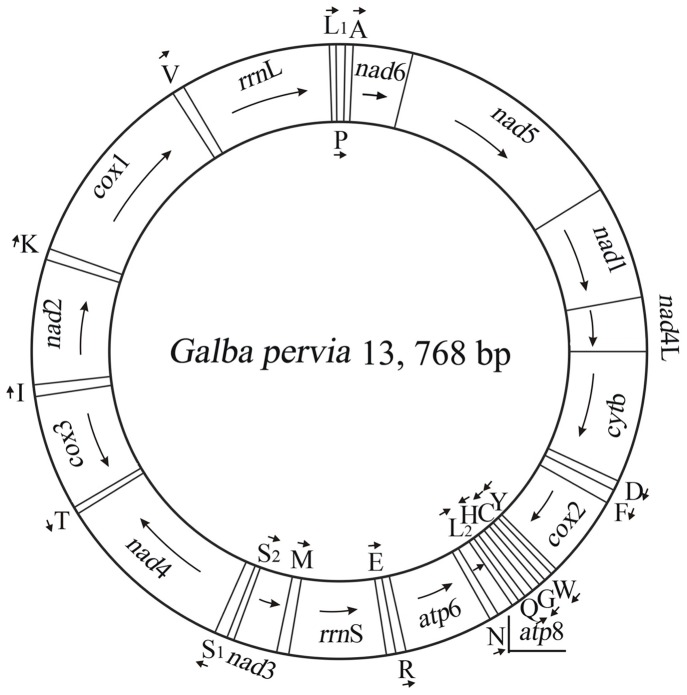
The mitochondrial genome of *Galba pervia*. Gene scaling is only approximate. All genes have standard nomenclature including the 22 tRNA genes, which are designated by the one-letter code for the corresponding amino acid, with numerals differentiating each of the two leucine- and serine-specifying tRNA (L1 and L2 for codon families CUN and UUR, respectively; S1 and S2 for codon families AGN and UCN, respectively).

The complete mt genome of *G. pervia* was 13,768 bp in length (Figure 1), and the mtDNA sequence was deposited in GenBank (accession number JN564796). The *G. pervia* mt genome contains 13 protein-coding genes (*cox*1-3, *nad*1-6, *nad*4L, *atp*6, *atp*8 and *cyt*b), a small subunit ribosomal RNA gene (*rrn*S), a large subunit ribosomal RNA gene (*rrn*L), and 22 transfer RNA genes, but without lengthy non-coding regions (Table 2). As found in other Gastropoda species, most of these genes are coded on the heavy strand (H-strand) except for *atp*6, *atp*8, *nad*3, *cox*3, 8 tRNA genes and *rrn*S. The details of gene locations were given in Table 2.

**Table 2:** Positions and nucleotide lengths of the mitochondrial genome of *Galba pervia*.

Gene	Position	Strand	Codon	Anticodon	Intergenic nucleotides^a^
	From-To (length)		Start/Stop		
*nad*5	1–1653 (1653)	H	ATA/TAG		+15
*nad*1	1669–2529 (861)	H	ATG/TAA		−1
*nad*4L	2529–2993 (465)	H	ATG/TAG		−158
*cyt*b	2836–3933 (1098)	H	ATA/TAG		−29
tRNA-Asp (D)	3905–3971 (67)	H		GTC	−9
tRNA-Phe (F)	3963–4025 (63)	H		GAA	+27
*cox*2	4053–4685 (633)	H	ATA/TAA		−19
tRNA-Tyr (Y)	4667–4726 (60)	H		TGT	−6
tRNA-Trp (W)	4721–4787 (67)	H		TCA	−8
tRNA-Cys (C)	4780–4842 (63)	H		GCA	+1
tRNA-Gly (G)	4844–4918 (75)	H		TCC	−19
tRNA-His (H)	4900–4959 (60)	H		GTG	+6
tRNA-Gln (Q)	4966–5029 (64)	L		TTG	−10
tRNA-Leu (UUR) (L2)	5020–5082(63)	L		TAA	−32
*atp*8	5051–5197 (147)	L	ATA/TAA		+30
tRNA-Asn (N)	5228–5292 (65)	L		GTT	−51
*atp*6	5242–5928 (687)	L	ATA/TAA		+3
tRNA-Arg (R)	5932–5996 (65)	L		TCG	−9
tRNA-Glu (E)	5988–6054 (67)	L		TTC	0
*rrn*S	6055–6767 (713)	L			+1
tRNA-Met (M)	6769–6835 (67)	L		CAT	−6
*nad*3	6830–7168 (339)	L	ATA/TAA		+16
tRNA-Ser UCN (S2)	7185–7247(63)	L		TGA	−7
tRNA-Ser AGN (S1)	7241–7294 (54)	H		GCT	+5
*nad*4	7310–8647 (1338)	H	ATA/TAA		−29
tRNA-Thr (T)	8619–8684 (66)	L		TGT	−20
*cox*3	8665–9462 (798)	L	ATG/TAA		+42
tRNA-Ile (I)	9505–9567(63)	H		GAT	+25
*nad*2	9593–10501(909)	H	ATA/TAA		−29
tRNA-Lys (K)	10473–10541 (69)	H		TTT	−29
*cox*1	10513–12057 (1545)	H	ATA/TAA		−2
tRNA-Val (V)	12056–12116 (61)	H		TAC	+3
*rrn*L	12120–13128 (1009)	H			−6
tRNA-Leu CUN (L1)	13123–13187 (65)	H		TAG	−5
tRNA-Pro (P)	13183–13246 (64)	H		TGG	−1
tRNA-Ala (A)	13246–13312 (67)	H		TGC	−41
*nad*6	13272–13763 (492)	H	ATG/TAA		+5

a indicates gap nucleotides (positive value) or overlapped nucleotides (negative value) between two adjacent genes.

The nucleotide compositions of the complete mtDNA sequence of *G. pervia* are biased toward A and T, with T being the most favored nucleotide and C the least favored, in accordance with the mt genome of *R. balthica* and *Biomphalaria glabrata* and *B. tenagophila* [22], [23]. The content of A+T is 72.67% for *G. pervia* (32.21% A, 40.46% T, 14.58% G and 12.75% C) (Table 3), 71.29% for *R. balthica* (31.68% A, 39.61% T, 15.39% G and 13.31% C), 75.78% for *B. tenagophila* (33.66% A, 42.12% T, 13.50% G and 10.72% C), respectively. Strand asymmetry (strand compositional bias) is usually reflected by skewness [24], which is calculated as (A%−T%)/(A%+T%) and (G%−C%)/(C%+G%), respectively. AT-skews and GC-skews of the whole mt genome were calculated for Pulmonata species to date (Table 3). This composition of full mtDNA sequence of *G. pervia* is strongly skewed away from A in favor of T (AT skew = −0.114), and GC skew = 0.067. The pattern of skew values of *G. pervia* is highly congruent with those observed in the mtDNA sequences of other pulmonate animals (Table 3). Previous studies suggested that GC skew is the best indicators of strand asymmetry [25]. Hence, all Pulmonata species in the present study show strand asymmetry (GC skew between −0.071and 0.210) (Table 3). Interestingly, in all the mt genome sequences of Pulmonata species reported to date, only the GC skew of *Rhopalocaulis grandidieri* has negative value due to that the C content of *R. grandidieri* mt genome is relatively higher than its G content. In mammals, these asymmetrical and biased base composition of mt genomes may be due to the spontaneous deamination process of C and A in the H-strand during replication [26], [27].

**Table 3:** Nucleotide composition of the mitochondrial genomes of Pulmonata species.

Species	Nucleotide frequency (%)				Whole genome sequence		
	A	T	G	C	A+T%	AT skew	GC skew
*Albinaria coerulea*	32.75	37.90	15.54	13.81	70.65	−0.073	0.059
*Ascobulla fragilis*	30.12	36.93	17.82	15.13	67.05	−0.102	0.082
*Biomphalaria glabrata*	33.05	41.58	14.08	11.29	74.63	−0.114	0.110
*Biomphalaria tenagophila*	33.66	42.12	13.50	10.72	75.78	−0.112	0.115
*Cepaea nemoralis*	26.16	33.63	21.26	18.94	59.79	−0.125	0.058
*Elysia chlorotica*	26.92	36.99	19.23	16.86	63.91	−0.158	0.066
*Myosotella myosotis*	23.67	31.35	23.63	21.35	55.02	−0.140	0.051
*Auriculinella bidentata*	25.83	30.88	22.65	20.41	56.71	−0.089	0.052
*Ovatella vulcani*	25.05	29.70	23.69	21.55	54.76	−0.085	0.047
*Pedipes pedipes*	28.59	33.73	19.27	18.42	62.31	−0.082	0.023
*Onchidella celtica*	25.26	34.06	21.77	18.92	59.31	−0.148	0.070
*Peronia peronii*	27.06	37.28	20.29	15.37	64.34	−0.159	0.138
*Platevindex mortoni*	27.27	35.72	20.23	16.78	62.99	−0.134	0.093
*Pyramidella dolabrata*	27.44	35.97	19.60	16.97	63.41	−0.135	0.072
*Siphonaria pectinata*	29.76	37.06	18.26	14.92	66.82	−0.109	0.101
*Siphonaria gigas*	24.32	37.24	23.35	15.08	61.56	−0.210	0.215
*Rhopalocaulis grandidieri*	29.27	33.88	17.11	19.73	63.15	−0.073	−0.071
*Salinator rhamphidia*	26.66	35.58	20.83	16.93	62.23	−0.143	0.103
*Succinea putris*	33.80	42.89	12.08	10.82	76.69	−0.119	0.055
*Trimusculus reticulatus*	26.40	34.72	20.64	18.24	61.12	−0.136	0.062
*Radix balthica*	31.68	39.61	15.39	13.31	71.29	−0.111	0.072
*Galba pervia*	32.21	40.46	14.58	12.75	72.67	−0.114	0.067

**Figure 2:**
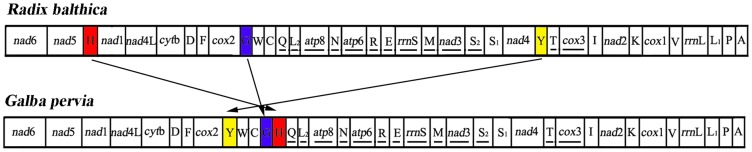
Comparison of the mitochondrial gene arrangement between *Galba pervia* and *Radix balthica*. All genes have standard nomenclature including the 22 tRNA genes, which are designated by the one-letter code for the corresponding amino acid, with numerals differentiating each of the two leucine- and serine-specifying tRNA (L1 and L2 for codon families CUN and UUR, respectively; S1 and S2 for codon families AGN and UCN, respectively). Underlined genes are coded on the light strand.

The gene arrangement differs among the mt genome sequences of 21 pulmonate animals, including that of *R. balthica* and *B. glabrata* and *B. tenagophila* (not shown). The gene arrangement in protein-coding genes seems to be stable in this group with a few exceptions. However, tRNA gene arrangement among these pulmonate animals is highly diversified, which provides further support for considerable variation in mt gene arrangement among pulmonate animals. Compared to the mt gene arrangement of *R. balthica*, the mt genome of *G. pervia* shows a novel gene arrangement (Figure 2). All rearranged genes are tRNA genes, in which 3 tRNA genes (tRNA-His, tRNA-Gly and tRNA-Tyr) changed their positions or directions (Figure 2). The tRNA-His arrangements represent translocation, tRNA-Gly arrangements represent shuffling, and tRNA-Tyr arrangements represent remote inversion translocations. Generally, tRNA gene rearrangements can be classified as translocations (across a protein-coding gene), local inversion (inverted but remaining in the position), remote inverted (translocated and inverted), and shuffling (on the same mt strand but in a different position) [28]. Within the Gastropoda, gene rearrangements have been particularly prevalent, and the mt gene rearrangment events in Gastropoda mt genomes have been discussed [29], [30]. To date, four mechanisms have been proposed to explain mt gene rearrangement in metazoan: (i) the tandem duplication followed by random loss (TDRL) of supernumerary genes owning to selection favoring small genomes [31], [32], (ii) illicit priming of replication by tRNA genes [33], (iii) tandem duplication followed by nonrandom loss of excess genes [34], and (iv) nonhomologous intergenome or intragenome recombination is presumed to be the most possible explanation for local inversion [35]. Considering all the data available so far, these mechanisms still do not explain mt gene rearrangment events in Gastropoda, because these compactly organized mt genomes of Gastropoda (with very few and short noncoding sequences) suggest strong selection against maintaining remnants of duplication events [36]. However, in *G. pervia*, a 6 bp and in *R. balthica* a 156 intergenic region were found in the location of tRNA-His, which might support TDRL as a mechanism acting in Pulmonata mt genome rearrangements.

Overlapping of adjacent genes (protein-encoding genes overlapped) is common in many animal mt genomes, although the extent of overlaps varies [37]–[40]. The mt genes of *G. pervia* overlap a total of 526 bp in 21 locations which range from 1 to 158 bp, including overlapping between protein-encoding and tRNA genes (Table 2) which has also been found in Gastropoda mt genomes [41], [42]. The *G. pervia* mt genes are separated by intergenic spacer sequences of a total of 179 bp in length, which are located in 13 regions and range from 1 to 42 bp in size (Table 2). The longest intergenic region (42 bp) is located between *cox*3 and tRNA-Ile genes.

### Protein-coding genes and codon usage patterns

The boundaries between protein-coding genes in the mt genome of *G. pervia* were determined by aligning their sequences and by identifying translation initiation and termination codons with comparison to those of *R. balthica* and *Biomphalaria* spp.. The predicted translation initiation and termination codons for the protein-coding genes of *G. pervia* mt genome were compared with those of *R. balthica* and *Biomphalaria* spp.. As shown in Table 2, the start codons of 13 protein-coding genes are ATN codon, which is typical of most metazoan mt genomes. The start codons inferred in the mt genome of *G. pervia* are ATA and ATG, and all reading-frames of the *G. pervia* ended with TAG or TAA as termination codons, whereas no anomalous initiation codons and incomplete stop codons are used, although they frequently occur in protein-coding genes of most Gastropoda mtDNA genomes [29].

The pattern of codon usage in the mtDNA of *G. pervia* was also studied. Excluding the termination codons, a total of 3655 amino acids are encoded by the *G. pervia* mt genome. Codons composed of A and T are more frequently used in protein-coding genes, which seems to reflect the high content of A+T in the entire mt genome of *G. pervia*. For example, the most frequently used amino acids were Leu (15.05%), followed by Phe (11.24%), Ser (10.76%) and IIe (8.34%) (Table 4).

**Table 4:** Codon usage of *Galba pervia* mitochondrial protein-coding genes.

Amino acid	Codon	Number	Frequency (%)	Amino acid	Codon	Number	Frequency (%)
Phe	TTT	374	10.23	Met	ATA	157	4.29
Phe	TTC	27	1.01	Met	ATG	45	1.23
Leu	TTA	377	10.31	Thr	ACT	89	2.43
Leu	TTG	48	1.31	Thr	ACC	12	0.32
Ser	TCT	107	2.92	Thr	ACA	56	1.53
Ser	TCC	13	0.35	Thr	ACG	6	0.16
Ser	TCA	92	2.51	Asn	AAT	118	3.22
Ser	TCG	15	0.41	Asn	AAC	26	0.71
Tyr	TAT	127	3.47	Lys	AAA	87	2.38
Tyr	TAC	25	0.68	Lys	AAG	10	0.27
Term	TAA	10	0.27	Ser	AGT	46	1.25
Term	TAG	3	0.08	Ser	AGC	12	0.32
Cys	TGT	45	1.23	Ser	AGA	86	2.35
Cys	TGC	10	0.27	Ser	AGG	24	0.65
Trp	TGA	65	1.77	Val	GTT	98	2.68
Trp	TGG	15	0.41	Val	GTC	9	0.24
Leu	CTT	68	1.86	Val	GTA	107	2.92
Leu	CTC	1	0.02	Val	GTG	27	0.73
Leu	CTA	52	1.42	Ala	GCT	85	2.32
Leu	CTG	5	0.13	Ala	GCC	16	0.43
Pro	CCT	70	1.91	Ala	GCA	67	1.83
Pro	CCC	6	0. 16	Ala	GCG	9	0.24
Pro	CCA	48	1.31	Asp	GAT	61	1.66
Pro	CCG	9	0.24	Asp	GAC	5	0.13
His	CAT	50	1.36	Glu	GAA	66	1.80
His	CAC	18	0.49	Glu	GAG	16	0.43
Gln	CAA	54	1.47	Gly	GGT	58	1.58
Gln	CAG	7	0.19	Gly	GGC	10	0.27
Arg	CGT	20	0.54	Gly	GGA	72	1.96
Arg	CGC	1	0.02	Gly	GGG	61	1.66
Arg	CGA	35	0.95	IIe	ATT	275	7.52
Arg	CGG	3	0.05	IIe	ATC	39	0.82

Total number of codons is 3655.

Term = Stop codon.

### Transfer RNA and ribosomal RNA genes

The 22 tRNA genes in *G. pervia* mt genome vary in length from 53 to 75 nucleotides with differences in stem and loop sizes of dihydrouridine (D) and TΨC loops. The 22 tRNA genes are located on both strands. Of these, 14 tRNA are encoded on the H-strand and 8 on the light strand (L-strand) (Table 2). The order and orientation of the gene arrangement pattern are identical to that of *R. balthica*, except for the positions and directions of the tRNA-His, tRNA-Gly and tRNA-Tyr genes. All of the 22 tRNA genes can be folded into normal cloverleaf structure, except for tRNA-Ser^(AGN)^ that lacks DHU arm. Their putative secondary structures are similar to those of *R. balthica* or *B. tenagophila* (not shown), indicating their similar functions. In mt genomes of most Gastropoda animals, tRNA-Ser^(UCN)^ generally lacks DHU arm [30], [42], [43], but it has a standard cloverleaf structure in mt genome of *G. pervia*. It is interesting that the secondary structures of mt tRNA genes are highly variable among Gastropoda animals. Previous studies suggested that the reduction of tRNA stem was caused by a strong pressure for mt genome minimization [44].

The *rrn*S and *rrn*L genes of *G. pervia* were identified by sequence comparison with those of *R. balthica*. The *rrn*S is located between tRNA-Glu and tRNA-Met, and the *rrn*L is located between tRNA-Val and the tRNA-Leu^CUN^. The lengths of the *rrn*S and *rrn*L genes of *G. pervia* are 713 bp and 1009 bp, respectively. The lengths of the *rrn*S and *rrn*L genes of *R. balthica* are 713 bp and 969 bp, respectively. The A+T contents of the *rrn*S and *rrn*L of *G. pervia* are 72.09% and 74.93%, respectively. Sequence identities in the *rrn*S and *rrn*L genes between *G. pervia* and *R. balthica* are 83.45% and 81.97%, respectively.

### Phylogenetic analyses

**Figure 3:**
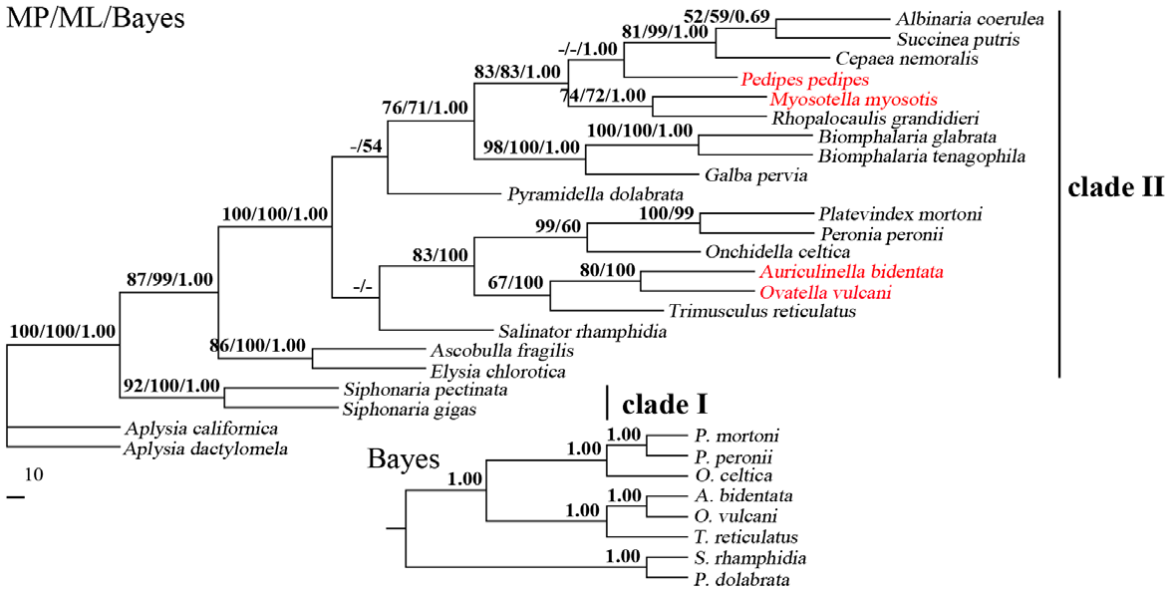
Inferred phylogenetic relationship among Pulmonata species based on mitochondrial DNA sequences. The concatenated amino acid sequences of 13 protein-coding genes were analyzed utilizing maximum parsimony (MP), maximum likelihood (ML) and Bayesian analysis (Bayes), using *Aplysia californica* and *A. dactylomela* as outgroup. The numbers along branches indicate bootstrap probability (BP) and posterior probability (PP) resulting from different analyses in the order: MP/ML/Bayes. Alternative topologies were correspondingly shown on the downside of the tree. The colourred species indicated that the phylogenetic status of the family Ellobiidae remains problematic.

The phylogenetic relationships of 20 Pulmonata species based on concatenated amino acid sequence datasets, plus the mt DNA sequence of *G. pervia* obtained in the present study, using maximum parsimony (MP), maximum likelihood (ML) and Bayesian analyses (Bayes) analyses are shown in Figure 3. The amino acid sequences of *R. balthica* were not used due to its low quality mt genome. The topologies of the trees from the MP, ML and Bayes analyses were identical or similar. In the tree, two major clades were recovered within Pulmonata: clade I and clade II form monophyletic groups, respectively. Within the clade I, *Siphonaria pectinata* and *S. gigas* clustered together with high statistical support, indicating that *S. pectinata* is sister to *S. gigas*. Within the clade II, Placobranchidae+Volvatellidae and other families form monophyletic groups, respectively.

The results of the present study revealed that *G. pervia* and *B. tenagophila* are closely related with high statistical support, indicating that Lymnaeidae and Planorbidae are closely related families, consistent with previous classification based on their morphological features and molecular data [45], [46]. Interestingly, MP and ML analyses revealed that families Amphibolidae and Pyramidellidae are not closely related (with very low bootstrap probability value), whereas Bayes analysis strongly supported that Amphibolidae and Pyramidellidae are closely related with high posterior probability value (Figure 3). Recent molecular phylogenetic studies revealed that the monophy of Pulmonata was clearly rejected by the phylogenetic position of a representative of the Systellommatophora (*Onchidella celtica*) which is more closely related to *Pyramidella dolabrata* than it was to any other Pulmonate [47]. This is probably due to the use of a small dataset. Thus, sampling of more taxa is needed to more accurately define phylogenetic positions of these families in further studies. In the present study, the phylogenetic status of the family Ellobiidae remains problematic, consistent with findings of recent molecular phylogenetic studies [48], [49].

Here, we will not further discuss the phylogenetic relationships of these families (eg., Clausiliidae, Aplysiidae, Volvatellidae, Pleurobranchidae, Siphonariidae and Ellobiidae) since their phylogenetic relationships have been discussed in detail by a recent study using nearly 80 Pulmonata species based on analyses of 18S, 16S and CO1 sequences [50]. Some recent studies showed that analyses using whole mt genome sequences (amino acid sequences) yield more accurate results than that using singe or small sets of gene sequences [51], [52]. Thus, more mt genomes of Pulmonata need to be sequenced and evolutionary relationships of Pulmonata should be reexamined.

In conclusion, the present study determined the mt genome sequence of *G. pervia*, which represents the first sequenced high quality mt genome of freshwater snails of the family Lymnaeidae. The *G. pervia* mt genome exhibits novel mt gene arrangement compared with other Pulmonata species. Phylogenetic analysis based on the mt amino acid sequence dataset revealed that Lymnaeidae and Planorbidae are closely related families, supporting previous classifications based on their morphology and molecular studies. These novel mtDNA data should be useful for further studying the population genetics and phylogeographics of this freshwater snail, which in turn would contribute to the effective control of *Fasciola* spp. transmitted by it.

## Materials and Methods

### Sample collection and DNA extraction

An adult freshwater snail representing *G. pervia* was collected from Guilin City, Guangxi Zhuang Nationality Autonomous Region, China. The specimen was washed in physiological saline, identified morphologically to species according to existing keys and descriptions [53], fixed in 70% (v/v) ethanol and stored at −20°C until use.

Total genomic DNA was extracted from its thoracic muscle tissue by treatment with sodium dodecyl sulphate/proteinase K (Merck), followed by purification using Wizard™ DNA Clean-Up System (Promega) and then eluted into 60 µl H_2_O according to the manufacturer's recommendations. The DNA sample was stored at −20°C until further use.

### Amplification and sequencing of partial *cox*1, *nad*1, *cyt*b, *cox*3 and *rrn*L

Partial fragments of mt *cox*1, *nad*1, *cyt*b, *cox*3 and *rrn*L genes were amplified using 5 sets of primers (Table 5) designed according to mtDNA sequences of other Pulmonata deposited in GenBank. PCR reactions were carried out in a 25 µl reaction volume consisting of 16.75 µl sterile deionized water, 2.5 µl 10×PCR Buffer (Mg^2+^ free), 2.0 µl MgCl_2_ (25 mM), 2.0 µl dNTPs (2.5 mM each), 0.25 µl each primer (50 pmol/µl), 0.25 µl ExTaq DNA polymerase (5 U/µl, Takara) and 1.0 µl DNA template (40 ng/µl) under the following conditions: after an initial denaturation at 94°C for 5 min, then 94°C for 1 min (denaturation), 50–55°C for 30 s (annealing), 72°C for 1 min (extension) for 35 cycles, followed by 72°C for 10 min (final extension). Each amplicon (5 µL) was examined by agarose gel electrophoresis to validate amplification efficiency. Then the *cox*1, *nad*1, *cyt*b, *cox*3 and *rrn*L amplicons were sent to BGI-Shenzhen Company, China for sequencing from both directions.

**Table 5:** Primers used to amplify short-PCR fragments from *Galba pervia*.

Name of primer	Sequence (5′ to 3′)
GP*cox*1F	TCTTTTRTTCTTCTTTTATGTTC
GP*cox*1R	ATTRAAATTTCGATCTGTTAA
GP*rrn*LF	CGGCCGCCTGTTTATCAAAAACAT
GP*rrn*LR	GGAGCTCCGGTTTGAACTCAGATC
GP*nad*1F	GAACGAAAAATTTTAGGKTATRTTCAAATTCG
GP*nad*1R	ATTAAAACCAGAAACTAAT
GP*cyt*bF	TTTCTTTCTATACAYTATACA
GP*cyt*bR	TAWGGTTYTTCAATAGGACA
GP*cox*3F	ACTACATCAACRAAATGTCARTATCA
Gp*cox*3R	CCTTTTCAYTTAGTAGAAT

### Long-PCR amplification and sequencing

The obtained nucleotide sequences of partial *cox*1, *nad*1, *cyt*b, *cox*3 and *rrn*L were used to design primer sets (Table 6) for long PCR amplification of the entire *G. pervia* mt genome. Five overlapping long PCR fragments covering the entire mt genome of *G. pervia* were obtained. The Long-PCR reaction volume amounted 50 µl containing 27.5 µl sterile deionized water, 5.0 µl 10×LA PCR Buffer (Mg^2+^ free), 5.0 µl MgCl_2_ (25 mM), 8.0 µl dNTPs (2.5 mM each), 0.5 µl each primer (50 pmol/µl), 0.5 µl LA *Taq* DNA polymerase (5 U/µl, Takara) and 3 µl DNA template (40 ng/µl). Long-PCR cycling conditions used were 92°C for 2 min (initial denaturation), then 92°C for 10 s (denaturation), 40°C for 30 s (annealing), and 60°C for 5 min (extension) for 5 cycles, followed by 92°C for 10 s, 40°C for 30 s, and 66°C for 5 min for 20 cycles and a final extension at 66°C for 10 min. All amplifications were done on a T-Gradient thermocycler (Biometra, Germany). The 5 long-PCR fragments were sequenced using a primer-walking strategy.

**Table 6:** Primers used to amplify Long-PCR fragments from *Galba pervia*.

Name of primer	Sequence (5′ to 3′)
GP*cox*1u1	TCTTTTRTTCTTCTTTTATGTTC
GP*rrn*Ld1	GGAGCTCCGGTTTGAACTCAGATC
GP*rrn*Lu2	CGGCCGCCTGTTTATCAAAAACAT
GP*nad*1d2	ATTAAAACCAGAAACTAAT
GP*nad*1u3	GAACGAAAAATTTTAGGKTATRTTCAAATTCG
GP*cyt*bd3	TAWGGTTYTTCAATAGGACA
GP*cyt*bu4	TTTCTTTCTATACAYTATACA
GP*cox*3d4	CCTTTTCAYTTAGTAGAAT
GP*cox*3u5	ACTACATCAACRAAATGTCARTATCA
GP*cox*1d5	ATT RAA ATT TCG ATC TGT TAA

### Gene annotation and sequence analysis

Sequences were assembled manually and aligned against the complete mt genome sequence of *R. balthica* and *B. tenagophila* using the computer program Clustal X 1.83 [54] to identify gene boundaries. The open-reading frames and codon usage profiles of protein-coding genes were analysed by the Open Reading Frame Finder (http://www.ncbi.nlm.nih.gov/gorf/gorf.html) using the invertebrate mitochondrial code. Translation initiation and translation termination codons were identified based on comparison with the mt genome of *R. balthica* and *B. tenagophila*. The amino acid sequences inferred for the mt genes of *G. pervia* were aligned with those of *R. balthica* and *B. tenagophila* by using Clustal X 1.83. Based on pairwise comparison, amino acid identity (%) was calculated for homologous genes. Codon usage was examined based on the relationships between the nucleotide composition of codon families and amino acid occurrence, where the genetic codons are partitioned into AT rich codons, GC-rich codons and unbiased codons. For analyzing ribosomal RNA genes, putative secondary structures of 22 tRNA genes were identified using tRNAscan-SE [55], of the 22 tRNA genes, 5 were identified using tRNAscan-SE, the other 17 tRNA genes were found by eye inspection, and rRNA genes were identified by comparison with the mt genome of *R. balthica* and *B. tenagophila*.

### Phylogenetic analyses

Phylogenetic relationship among the 20 Pulmonata species (Table 1), plus the mt DNA sequence of *G. pervia* obtained in the present study was reconstructed based on amino acid sequences of 13 protein-coding genes using the 2 Opisthobranchia species (*Aplysia californica*, GenBank accession number NC_005827 and *A. dactylomela*, NC_015088) as the outgroup. Each gene was translated into amino acid sequence using the invertebrate mitochondrial genetic code in MEGA 4 [56], and aligned based on its amino acid sequence using default settings, and ambiguously aligned regions were excluded using Gblocks online server (http://molevol.cmima.csic.es/castresana/Gblocks_server.html) [57] using the options for a less stringent selection. The final amino acid sequences of the 13 protein-coding genes were then concatenated into single alignments for phylogenetic analyses. Three different inference methods, namely MP, ML, and Bayes, were used for phylogenetic analyses. MP analysis was performed using PAUP* 4.0b10 [58], with indels treated as missing character states. A total of 1,000 random addition searches using TBR were performed for each MP analysis. Bootstrap probability (BP) was calculated from 1,000 bootstrap replicates with 10 random additions per replicate in PAUP. ML analyses were performed using PhyML 3.0 [59], and the MtArt+I+G+F model with its parameter for the concatenated dataset was determined for the ML analysis using ProtTest 10.2 based on the Akaike information criterion (AIC) [60]. BP value for ML trees was calculated using 1000 bootstrap replicates. Bayesian analyses were conducted with four independent Markov chains run for 1,000,000 metropolis-coupled MCMC generations, sampling a tree every 100 generations in MrBayes 3.1.1 [61]. The first 2,500 trees were omitted as burn-in and the remaining trees were used to calculate Bayesian posterior probabilities (PP). Phylograms were drawn using the Tree View program version 1.65 [62].

## References

[1] Relf V, Good B, Hanrahan JP, McCarthy E, Forbes AB, et al (2011) Temporal studies on *Fasciola hepatica* in *Galba truncatula* in the west of Ireland. Vet Parasitol 175: 287–292. 21111536 10.1016/j.vetpar.2010.10.010

[2] Kaset C, Eursitthichai V, Vichasri-Grams S, Viyanant V, Grams R (2010) Rapid identification of lymnaeid snails and their infection with *Fasciola gigantica* in Thailand. Exp Parasitol 126: 482–488. 20685274 10.1016/j.exppara.2010.05.021

[3] Zhang HY, Kraemer F, Shen YL, Gu YF, Shen T, et al (1999) Detection of *Fasciola hepatica* in *galba pervia* by dot hybridization. Chin j vet parastiol 1: 1–3 (in Chinese).

[4] Ai L, Weng YB, Elsheikha HM, Zhao GH, Alasaad S, et al (2011) Genetic diversity and relatedness of *Fasciola* spp. isolates from different hosts and geographic regions revealed by analysis of mitochondrial DNA sequences. Vet Parasitol 181: 329–334. 21524854 10.1016/j.vetpar.2011.03.057

[5] Alasaad S, Soriguer RC, Abu-Madi M, El Behairy A, Jowers MJ, et al (2011) A TaqMan real-time PCR-based assay for the identification of *Fasciola* spp. Vet Parasitol 179: 266–271. 21334813 10.1016/j.vetpar.2011.01.059

[6] Lin RQ, Dong SJ, Nie K, Wang CR, Song HQ, et al (2007) Sequence analysis of the first internal transcribed spacer of rDNA supports the existence of the intermediate *Fasciola* between *F. hepatica* and *F. gigantica* in mainland China. Parasitol Res 101: 813–817. 17356892 10.1007/s00436-007-0512-0

[7] Spithill TW, Dalton JP (1998) Progress in development of liver fluke vaccines. Parasitol Today 14: 224–228. 17040765 10.1016/s0169-4758(98)01245-9

[8] Mas-Coma S, Bargues MD, Valero MA (2005) Fascioliasis and other plant-borne trematode zoonoses. Int J Parasitol 35: 1255–1278. 16150452 10.1016/j.ijpara.2005.07.010

[9] Wolstenholme DR (1992) Animal mitochondrial DNA, structure and evolution. Int Rev Cytol 141: 173–216. 1452431 10.1016/s0074-7696(08)62066-5

[10] Li MW, Lin RQ, Song HQ, Wu XY, Zhu XQ (2008) The complete mitochondrial genomes for three *Toxocara* species of human and animal health significance. BMC Genomics 9: e224. 10.1186/1471-2164-9-224 PMC2396643 18482460

[11] Liu GH, Lin RQ, Li MW, Liu W, Liu Y, et al (2011) The complete mitochondrial genomes of three cestode species of *Taenia* infecting animals and humans. Mol Biol Rep 38: 2249–2256. 20922482 10.1007/s11033-010-0355-0

[12] Margam VM, Coates BS, Hellmich RL, Agunbiade T, Seufferheld MJ, et al (2011) Mitochondrial genome sequence and expression profiling for the legume pod borer *Maruca vitrata* (Lepidoptera: Crambidae). PLoS One 6: e16444. 21311752 10.1371/journal.pone.0016444 PMC3032770

[13] Tan HW, Liu GH, Dong X, Lin RQ, Song HQ, et al (2011) The Complete Mitochondrial Genome of the Asiatic Cavity-nesting Honeybee *Apis cerana* (Hymenoptera: Apidae). PLoS One 6: e23008. 21857981 10.1371/journal.pone.0023008 PMC3155526

[14] Wei SJ, Shi M, Chen XX, Sharkey MJ, van Achterberg C, et al (2010) New views on strand asymmetry in insect mitochondrial genomes. PLoS One 5: e12708. 20856815 10.1371/journal.pone.0012708 PMC2939890

[15] Lin RQ, Qiu LL, Liu GH, Wu XY, Weng YB, et al (2011) Characterization of the complete mitochondrial genomes of five *Eimeria* species from domestic chickens. Gene 480: 28–33. 21402132 10.1016/j.gene.2011.03.004

[16] Kurabayashi A, Ueshima R (2000) Complete sequence of the mitochondrial DNA of the primitive opisthobranch gastropod *Pupa strigosa*: systematic implication of the genome organization. Mol Biol Evol 17: 266–277. 10677849 10.1093/oxfordjournals.molbev.a026306

[17] Hoffmann RJ, Boore JL, Brown WM (1992) A novel mitochondrial genome organization for the blue mussel, *Mytilus edulis* . Genetics 131: 397–412. 1386586 10.1093/genetics/131.2.397 PMC1205014

[18] Ren J, Shen X, Jiang F, Liu B (2010) The mitochondrial genomes of two scallops, *Argopecten irradians* and *Chlamys farreri* (Mollusca: Bivalvia): the most highly rearranged gene order in the family Pectinidae. J Mol Evol 70: 57–68. 20013337 10.1007/s00239-009-9308-4

[19] Boettger C (1955) Die Taxonomie der euthyneuren Schnecken. Zool Anz 18: 253–280.

[20] Grande C, Templado J, Cervera JL, Zardoya R (2002) The complete mitochondrial genome of the Nudibranch *Roboastra europaea* (Mollusca: Gastropoda) supports the monophyly of Opisthobranchs. Mol Biol Evol 19: 1672–1685. 12270894 10.1093/oxfordjournals.molbev.a003990

[21] Boore JL (1999) Animal mitochondrial genomes. Nucleic Acids Res 27: 1767–1780. 10101183 10.1093/nar/27.8.1767 PMC148383

[22] Feldmeyer B, Hoffmeier K, Pfenninger M (2010) The complete mitochondrial genome of *Radix balthica* (Pulmonata, Basommatophora), obtained by low coverage shot gun next generation sequencing. Mol Phylogenet Evol 57: 1329–1333. 20875865 10.1016/j.ympev.2010.09.012

[23] DeJong RJ, Emery AM, Adema CM (2004) The mitochondrial genome of *Biomphalaria glabrata* (Gastropoda: Basommatophora), intermediate host of *Schistosoma mansoni* . J Parasitol 90: 991–997. 15562597 10.1645/GE-284R

[24] Perna NT, Kocher TD (1995) Patterns of nucleotide composition at fourfold degenerate sites of animal mitochondrial genomes. J Mol Evol 41: 353–358. 7563121 10.1007/BF00186547

[25] Hassanin A, Léger N, Deutsch J (2005) Evidence for multiple reversals of asymmetric mutational constraints during the evolution of the mitochondrial genome of metazoa, and consequences for phylogenetic inferences. Syst Biol 54: 277–298. 16021696 10.1080/10635150590947843

[26] Yang JS, Nagasawa H, Fujiwara Y, Tsuchida S, Yang WJ (2008) The complete mitochondrial genome sequence of the hydrothermal vent galatheid crab *Shinkaia crosnieri* (Crustacea: Decapoda: Anomura): a novel arrangement and incomplete tRNA suite. BMC Genomics 9: 257. 18510775 10.1186/1471-2164-9-257 PMC2442616

[27] Torricelli G, Carapelli A, Convey P, Nardi F, Boore JL, et al (2010) High divergence across the whole mitochondrial genome in the “pan-Antarctic” springtail *Friesea grisea*: evidence for cryptic species? Gene 449: 30–40. 19782734 10.1016/j.gene.2009.09.006

[28] Wei SJ, Shi M, Sharkey MJ, Achterberg C, Chen XX (2010) Comparative mitogenomics of Braconidae (Insecta: Hymenoptera) and the phylogenetic utility of mitochondrial genomes with special reference to Holometabolous insects. BMC Genomics 11: 371. 20537196 10.1186/1471-2164-11-371 PMC2890569

[29] Rawlings TA, MacInnis MJ, Bieler R, Boore JL, Collins TM (2010) Sessile snails, dynamic genomes: gene rearrangements within the mitochondrial genome of a family of caenogastropod mollusks. BMC Genomics 11: 440. 20642828 10.1186/1471-2164-11-440 PMC3091637

[30] Knudsen B, Kohn AB, Nahir B, McFadden CS, Moroz LL (2006) Complete DNA sequence of the mitochondrial genome of the sea-slug, *Aplysia californica*: conservation of the gene order in Euthyneura. Mol Phylogenet Evol 38: 459–469. 16230032 10.1016/j.ympev.2005.08.017

[31] Ki JS, Park HG, Lee JS (2009) The complete mitochondrial genome of the cyclopoid copepod *Paracyclopina nana*: a highly divergent genome with novel gene order and atypical gene numbers. Gene 435: 13–22. 19393182 10.1016/j.gene.2009.01.005

[32] Dowton M, Castro LR, Campbell SL, Bargon SD, Austin AD (2003) Frequent mitochondrial gene rearrangements at the hymenopteran nad3-nad5 junction. J Mol Evol 56: 517–526. 12698290 10.1007/s00239-002-2420-3

[33] Cantatore P, Gadaleta MN, Roberti M, Saccone C, Wilson AC (1987) Duplication and remoulding of tRNA genes during the evolutionary rearrangement of mitochondrial genomes. Nature 329: 853–855. 3670390 10.1038/329853a0

[34] Lavrov DV, Boore JL, Brown WM (2002) Complete mtDNA sequences of two millipedes suggest a new model for mitochondrial gene rearrangements: duplication and nonrandom loss. Mol Biol Evol 19: 163–169. 11801744 10.1093/oxfordjournals.molbev.a004068

[35] Fahrein K, Talarico G, Braband A, Podsiadlowski L (2007) The complete mitochondrial genome of *Pseudocellus pearsei* (Chelicerata: Ricinulei) and a comparison of mitochondrial gene rearrangements in Arachnida. BMC Genomics 8: 386. 17961221 10.1186/1471-2164-8-386 PMC2231378

[36] Grande C, Templado J, Zardoya R (2008) Evolution of gastropod mitochondrial genome arrangements. BMC Evol Biol 8: 61. 18302768 10.1186/1471-2148-8-61 PMC2291457

[37] Cai XQ, Liu GH, Song HQ, Wu CY, Zou FC, et al (2012) Sequences and gene organization of the mitochondrial genomes of the liver flukes *Opisthorchis viverrini* and *Clonorchis sinensis* (Trematoda). Parasitol Res 110: 235–243. 21626421 10.1007/s00436-011-2477-2

[38] Yasuda N, Hamaguchi M, Sasaki M, Nagai S, Saba M, et al (2006) Complete mitochondrial genome sequences for Crown-of-thorns starfish *Acanthaster planci* and *Acanthaster brevispinus* . BMC Genomics 7: 17. 16438737 10.1186/1471-2164-7-17 PMC1382216

[39] Catanese G, Manchado M, Infante C (2010) Evolutionary relatedness of mackerels of the genus *Scomber* based on complete mitochondrial genomes: strong support to the recognition of Atlantic *Scomber colia*s and Pacific *Scomber japonicus* as distinct species. Gene 452: 35–43. 20035845 10.1016/j.gene.2009.12.004

[40] Lei R, Shore GD, Brenneman RA, Engberg SE, Sitzmann BD, et al (2010) Complete sequence and gene organization of the mitochondrial genome for Hubbard's sportive lemur (*Lepilemur hubbardorum*). Gene 464: 44–49. 20547216 10.1016/j.gene.2010.06.001

[41] Cunha RL, Grande C, Zardoya R (2009) Neogastropod phylogenetic relationships based on entire mitochondrial genomes. BMC Evol Biol 9: 210. 19698157 10.1186/1471-2148-9-210 PMC2741453

[42] Bandyopadhyay PK, Stevenson BJ, Cady MT, Olivera BM, Wolstenholme DR (2006) Complete mitochondrial DNA sequence of a Conoidean gastropod, *Lophiotoma (Xenuroturris) cerithiformis*: gene order and gastropod phylogeny. Toxicon 48: 29–43. 16806344 10.1016/j.toxicon.2006.04.013

[43] Dayrat B, Tillier A, Lecointre G, Tillier S (2001) New clades of euthyneuran gastropods (Mollusca) from 28S rRNA sequences. Mol Phylogenet Evol 19: 225–235. 11341805 10.1006/mpev.2001.0926

[44] Yamazaki N, Ueshima R, Terrett JA, Yokobori S, Kaifu M, et al (1997) Evolution of pulmonate gastropod mitochondrial genomes: comparisons of gene organizations of Euhadra, Cepaea and Albinaria and implications of unusual tRNA secondary structures. Genetics 145: 749–758. 9055084 10.1093/genetics/145.3.749 PMC1207859

[45] Paraense WL (2003) Planorbidae, lymnaeidae and physidae of Peru (Mollusca: Basommatophora). Mem Inst Oswaldo Cruz 98: 767–771. 14595453 10.1590/s0074-02762003000600010

[46] Kocot KM, Cannon JT, Todt C, Citarella MR, Kohn AB, et al (2011) Phylogenomics reveals deep molluscan relationships. Nature 477: 452–456. 21892190 10.1038/nature10382 PMC4024475

[47] Grande C, Templado J, Cervera JL, Zardoya R (2004) Molecular phylogeny of Euthyneura (Mollusca: Gastropoda). Mol Biol Evol 21: 303–313. 14660702 10.1093/molbev/msh016

[48] Klussmann-Kolb A, Dinapoli A, Kuhn K, Streit B, Albrecht C (2008) From sea to land and beyond–new insights into the evolution of euthyneuran Gastropoda (Mollusca). BMC Evol Biol 8: 57. 18294406 10.1186/1471-2148-8-57 PMC2287175

[49] White TR, Conrad MM, Tseng R, Balayan S, Golding R, et al (2011) Ten new complete mitochondrial genomes of pulmonates (Mollusca: Gastropoda) and their impact on phylogenetic relationships. BMC Evol Biol 11: 295. 21985526 10.1186/1471-2148-11-295 PMC3198971

[50] Dayrat B, Conrad M, Balayan S, White TR, Albrecht C, et al (2011) Phylogenetic relationships and evolution of pulmonate gastropods (Mollusca): new insights from increased taxon sampling. Mol Phylogenet Evol 59: 425–437. 21352933 10.1016/j.ympev.2011.02.014

[51] Whelan S, Goldman N (2001) A general empirical model of protein evolution derived from multiple protein families using a maximum-likelihood approach. Mol Biol Evol 18: 691–699. 11319253 10.1093/oxfordjournals.molbev.a003851

[52] Yang Z, Bielawski JP (2000) Statistical methods for detecting molecular adaptation. Trends Ecol Evol 15: 496–503. 11114436 10.1016/S0169-5347(00)01994-7 PMC7134603

[53] Liu YY, Wang YX (1965) Morphological and characteristic of Lymnaeidae. Bulletin Biol 3: 8–12 (in Chinese)..

[54] Thompson JD, Gibson TJ, Plewniak F, Jeanmougin F, Higgins DG (1997) The Clustal X windows interface: flexible strategies for multiple sequence alignment aided by quality analysis tools. Nucleic Acids Res 24: 4876–4882. 10.1093/nar/25.24.4876 PMC147148 9396791

[55] Lowe TM, Eddy SR (1997) tRNAscan-SE: A program for improved detection of transfer RNA genes in genomic sequence. Nucleic Acids Res 25: 955–964. 9023104 10.1093/nar/25.5.955 PMC146525

[56] Tamura K, Dudley J, Nei M, Kumar S (2007) MEGA4: molecular evolutionary genetics analysis (MEGA) software version 4.0. Mol Biol Evol 24: 1596–1599. 17488738 10.1093/molbev/msm092

[57] Talavera G, Castresana J (2007) Improvement of phylogenies after removing divergent and ambiguously aligned blocks from protein sequence alignments. Syst Biol 56: 564–577. 17654362 10.1080/10635150701472164

[58] Swofford DL (2002) Paup*: Phylogenetic Analysis Using Parsimony, version 4.0b10. Sinauer Associates, Sunderland, MA.

[59] Guindon S, Gascuel O (2003) A simple, fast, and accurate algorithm to estimate large phylogenies by maximum likelihood. Syst Biol 52: 696–704. 14530136 10.1080/10635150390235520

[60] Abascal F, Zardoya R, Posada D (2005) ProtTest: selection of best-fit models of protein evolution. Bioinformatics 21: 2104–2105. 15647292 10.1093/bioinformatics/bti263

[61] Ronquist F, Huelsenbeck JP (2003) MrBayes 3: Bayesian phylogenetic inference under mixed models. Bioinformatics 19: 1572–1574. 12912839 10.1093/bioinformatics/btg180

[62] Page RD (1996) TREEVIEW: an application to display phylogenetic trees on personal computers. Comput Appl Biosci 12: 357–358. 8902363 10.1093/bioinformatics/12.4.357

